# Inter‐ and Transgenerational Effects of Paternal Exposure to Inorganic Arsenic

**DOI:** 10.1002/advs.202002715

**Published:** 2021-02-18

**Authors:** Yingyun Gong, Yanfeng Xue, Xin Li, Zhao Zhang, Wenjun Zhou, Paola Marcolongo, Angiolo Benedetti, Shengyong Mao, Leng Han, Guolian Ding, Zheng Sun

**Affiliations:** ^1^ Department of Endocrinology and Metabolism The First Affiliated Hospital of Nanjing Medical University Nanjing 210029 China; ^2^ Division of Endocrinology Department of Medicine Baylor College of Medicine Houston TX 77030 USA; ^3^ National Center for International Research on Animal Gut Nutrition College of Animal Science and Technology Nanjing Agricultural University Nanjing 210095 China; ^4^ Department of Biochemistry and Molecular Biology McGovern Medical School University of Texas Health Science Center at Houston Houston TX 77030 USA; ^5^ Department of Molecular and Developmental Medicine University of Siena Siena 53100 Italy; ^6^ Obstetrics and Gynecology Hospital, Institute of Reproduction and Development, Fudan University Shanghai Key Laboratory of Embryo Original Diseases Shanghai 200011 China; ^7^ Department of Molecular and Cellular Biology Baylor College of Medicine Houston TX 77030 USA

**Keywords:** arsenic, diabetes, environmental health, epigenetic inheritance, metabolism, obesity

## Abstract

The rise of metabolic disorders in modern times is mainly attributed to the environment. However, heritable effects of environmental chemicals on mammalian offsprings' metabolic health are unclear. Inorganic arsenic (iAs) is the top chemical on the Agency for Toxic Substances and Disease Registry priority list of hazardous substances. Here, we assess cross‐generational effects of iAs in an exclusive male‐lineage transmission paradigm. The exposure of male mice to 250 ppb iAs causes glucose intolerance and hepatic insulin resistance in F1 females, but not males, without affecting body weight. Hepatic expression of glucose metabolic genes, glucose output, and insulin signaling are disrupted in F1 females. Inhibition of the glucose 6‐phosphatase complex masks the intergenerational effect of iAs, demonstrating a causative role of hepatic glucose production. F2 offspring from grandpaternal iAs exposure show temporary growth retardation at an early age, which diminishes in adults. However, reduced adiposity persists into middle age and is associated with altered gut microbiome and increased brown adipose thermogenesis. In contrast, F3 offspring of the male‐lineage iAs exposure show increased adiposity, especially on a high‐calorie diet. These findings have unveiled sex‐ and generation‐specific heritable effects of iAs on metabolic physiology, which has broad implications in understanding gene‐environment interactions.

## Graphical abstract

Inorganic arsenic (iAs) has sex‐ and generation‐specific heritable effects on metabolic physiology in mammals through the male germline lineage. Paternal exposure to iAs causes glucose intolerance and hepatic insulin resistance in F1 females, but not males. F2 offspring from grand paternal iAs exposure show reduced adiposity. F3 offspring of the male‐lineage iAs exposure show increased adiposity.

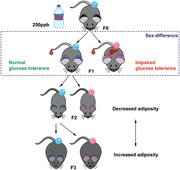

## 1 Introduction

The rise of metabolism‐related chronic diseases in industrialized society is mainly attributed to environmental factors because the population's genetic makeup cannot be drastically altered in such a short period. In addition to overnutrition and sedentary lifestyles, exposure to environmental chemicals can be an important factor. Industrialization in modern human history has been associated with widespread pollution of air, water, soil, and food.^[^ ^1^ ^]^ Many developed countries have seen a steady reduction of pollution in the past few decades,^[^ ^2^, ^3^ ^]^ but it is unclear how cross‐generational effects of environmental, chemical exposure in the descendants contribute to metabolic disorders later in life. Epigenetic inheritance is postulated to account for the long‐lasting effects of gene–environment (GxE) interactions.^[^ ^4^ ^]^ However, the transgenerational heritability of metabolic traits from environmental exposure is not rigorously tested in mammals.^[^ ^5^ ^]^

The maternal effects of environmental chemicals due to prenatal or in utero exposure have gained increasing interest, as recognized as the Developmental Origins of Health and Disease (DOHaD) paradigm.^[^ ^6^ ^]^ The effects observed under the maternal paradigm can be due to altered developmental processes or maternal nurturing behaviors rather than genuine epigenetic inheritance. Dissection of these effects takes specific experimental manipulations such as in vitro fertilization.^[^ ^7^ ^]^ By comparison, exposure to adult males followed by exclusive male‐lineage breeding has fewer confounding effects, which provides an efficient way to distinguish transgenerational effects from intergenerational effects.^[^ ^8^ ^]^ Although the paternal effects of dietary factors or psychological stress have been studied on F1 offspring,^[^ ^9^, ^10^, ^11^, ^12^ ^]^ it remains underexplored how paternal exposure to environmental chemicals affects metabolic health in F1 offspring. More importantly, the transgenerational effects of paternal exposures across multiple generations were unclear in mammals.

Inorganic arsenic (iAs) is the top chemical on the Agency for Toxic Substances and Disease Registry (ATSDR) priority list of hazardous substances. Over 200 million people worldwide drink water with iAs above the 10 ppb guideline established by World Health Organization (WHO) and United States Environmental Protection Agency (US EPA).^[^ ^13^, ^14^ ^]^ Source of contamination includes industrial or agricultural chemical waste and natural sources. iAs is found in the Earth's crust at 1.5–2 ppm and in the soil at 1–40 ppm. Here, we study the transgenerational effects of iAs on metabolic physiology. We exposed mice to 250 ppb in drinking water in the present study, which is comparable to human exposure in contaminated areas, especially when considering the human–mouse difference in iAs clearance.^[^ ^15^, ^16^, ^17^ ^]^

The transgenerational effects of iAs have not been studied in mammals, although the effect of early‐life iAs exposure on later‐life health has been characterized under the DOHaD paradigm.^[^ ^18^, ^19^, ^20^, ^21^, ^22^, ^23^, ^24^, ^25^, ^26^, ^27^, ^28^, ^29^, ^30^ ^]^ We focused on an exclusive male‐lineage exposure paradigm. This paradigm excludes most early‐life developmental processes from prenatal or in utero exposures,^[^ ^8^ ^]^ which allow us to study transgenerational effects within two generations.

We use the following principle in characterizing metabolic phenotypes in mice. We first examined body weight and adiposity. If there was no change in body weight, we further examined glucose tolerance, insulin secretion, and insulin sensitivity. However, if there was a change in body weight, we chose to focus on energy balance instead of glucose tolerance because any change in glucose tolerance was likely due to the altered adiposity and energy imbalance. As a result, we focused on the glucose tolerance phenotype in the F1 offspring, but adiposity and energy balance in the F2 and F3 generations.

## 2 Results

### 2.1 Paternal iAs Exposure Caused Sex‐Specific Effects on Glucose Metabolism in F1 Offspring

**Figure 1:**
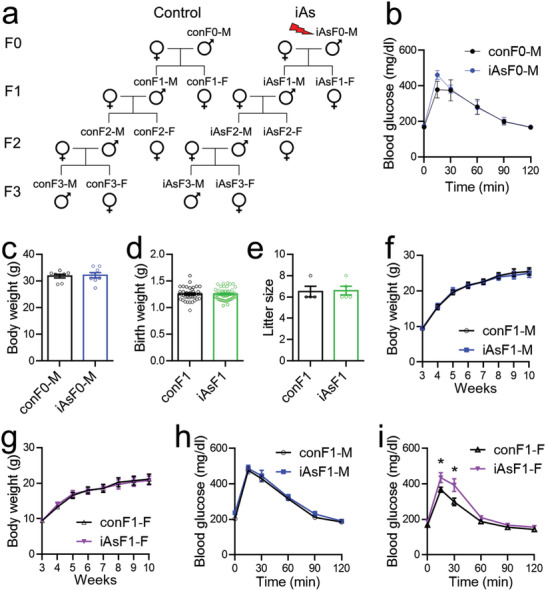
Paternal iAs exposure caused sex‐specific effects on glucose metabolism without altering body weight in the F1 offspring. a) Breeding scheme. b) Glucose tolerance test (GTT) in iAsF0 male at the time of breeding, *n* = 8 mice. c) Body weight of F0 males. d) Birth weight of the F1 offspring. e) Litter size of the F1 offspring. f,g) Body weight of F1 males and females, *n* = 9 mice. h,i) GTT in F1 offspring at 12 weeks old, *n* = 9 mice. Data are mean ± S.E.M. * *p* < 0.05 by *t*‐test or two‐way ANOVA with Holm–Sidak's posthoc.

To ensure that the intergenerational and transgenerational effects are from the male lineage, we used unrelated female breeders that were not exposed to iAs (**Figure** **1**a). We treated C57BL/6 male mice with iAs in drinking water at 250 ppb (corresponding to 3.325 × 10^−6^ m NaAsO_2_) for 3 weeks before breeding with unexposed female mice (Figure 1a). The breeding took place in one night in clean cages in the absence of iAs‐containing water, and the breeding females were quickly separated from breeding males after mating. The resultant F1 male (iAsF1‐M) mice were bred with unrelated, unexposed female mice to generate F2 offspring. The F2 male (iAsF2‐M) mice were then bred with unrelated, unexposed female mice to generate F3 offspring. The control groups were bred at the same time and were referred to as conF1, conF2, and conF3. None of the F1–3 offspring were ever directly exposed to iAs. The F0 male mice directly exposed to iAs (iAsF0‐M) showed normal body weight (Figure 1b) and glucose tolerance (Figure 1c) at the time of breeding, probably due to the short duration of iAs exposure. iAsF1 mice showed normal birth weight (Figure 1d), litter size (Figure 1e), and age‐dependent body weight gain on a normal chow diet (Figure 1f,g). iAsF1‐M mice showed normal glucose tolerance (Figure 1h). However, iAsF1 female (iAsF1‐F) mice showed persistent glucose intolerance (Figure 1i). Thus, paternal iAs exposure caused sex‐specific effects on glucose metabolism without altering body weight in the F1 offspring.

### 2.2 The Liver Plays a Central Role in the Glucose Intolerance in iAsF1 Females

**Figure 2:**
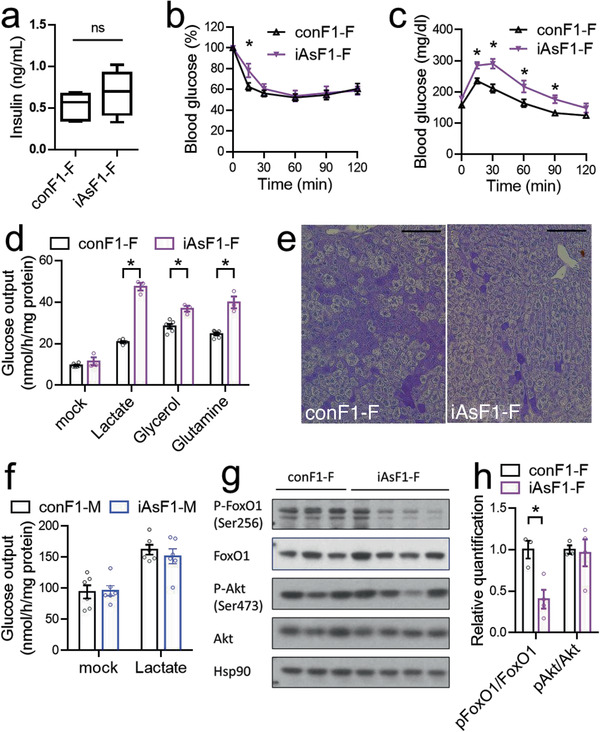
The liver plays a central role in the glucose phenotype in iAsF1‐F through an FoxO1‐related nuclear mechanism. a) Fasting serum insulin levels. Box plots center lines, limits, and whiskers represent the median, quartile, and minimum/maximum values, respectively, *n* = 8 mice. b) Insulin tolerance test (ITT) at 16 weeks old, *n* = 8 mice. c) Pyruvate tolerance test (PTT) at 18 weeks old, *n* = 8 mice. d) Glucose production assay in primary hepatocytes isolated from F1 females in the presence of the indicated substrates, *n* = 3 mice. e) Periodic acid–Schiff (PAS) staining of the liver in the fed condition. Scale bar = 100 µm. f) Glucose production assay in primary hepatocytes isolated from F1 males, *n* = 3 mice. g) Western blot analysis of molecular insulin signaling in the liver, *n* = 3–4 mice. h) Quantification of the western blot. Data are mean ± S.E.M. * *p* < 0.05 by two‐sided *t*‐test or two‐way ANOVA with Holm–Sidak's posthoc.

Female iAsF1 mice showed normal insulin levels (**Figure** **2**a), suggesting a potential insulin resistance that may underscore the glucose intolerance phenotype. Supporting this notion, iAsF1‐F mice had reduced insulin tolerance compared to conF1‐F (Figure 2b). Systemic insulin resistance can be due to impaired suppression of glucose production from the liver or impaired blood glucose clearance by muscle and adipose tissues. iAsF1‐F mice showed higher blood glucose levels than conF1‐F mice after treatment of pyruvate, a major gluconeogenic substrate (Figure 2c). This result suggests that the elevated hepatic glucose production mediates the glucose phenotype in iAsF1‐F mice. To further test the central role of the liver, we isolated primary hepatocytes and performed glucose output assays ex vivo. Glucose production from several precursors was increased in iAsF1‐F hepatocytes than conF1‐F (Figure 2d). Consistent with the higher glucose output, the iAsF1‐F liver showed a lower glycogen content than conF1‐F (Figure 2e). In contrast, primary hepatocytes from iAsF1‐M mice showed normal glucose output (Figure 2f), which is consistent with normal glucose tolerance in iAsF1‐M in vivo. Insulin is a major regulator of hepatic glucose production. Insulin suppresses hepatic gluconeogenesis by phosphorylating and activating AKT through the upstream cytosolic signaling events involving the insulin receptor and PI3K. AKT then phosphorylates transcriptional factor FoxO1 and inhibits its transactivation activity in the nucleus. Western blot analysis revealed unaltered AKT phosphorylation but reduced FoxO1 phosphorylation in the iAsF1‐F liver (Figure 2g,h). These results suggest a liver‐centric, FoxO1‐related, nuclear mechanism underpinning the glucose phenotype in iAsF1‐F mice.

### 2.3 Hepatic FoxO1/G6pc Signaling Contributes to the Enhanced Gluconeogenesis in iAsF1 Females

**Figure 3:**
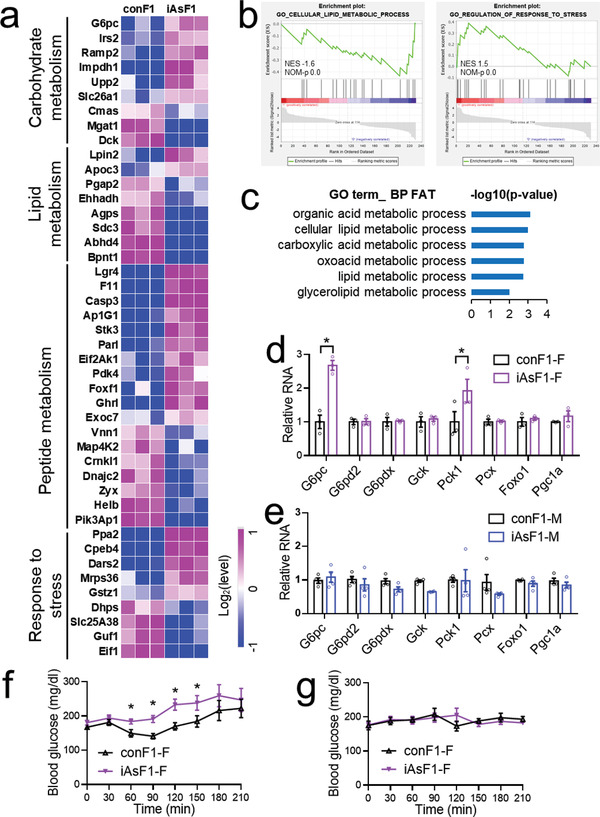
Hepatic FoxO1/G6pc signaling contributes to the enhanced gluconeogenesis in iAsF1‐F mice. a) A heat map of differentially expressed genes (DEGs) between female iAsF1 and conF1 livers in the refeeding condition. b) GSEA gene ontology (GO) enrichment of the DEGs. NES, normalized enrichment score. NOM‐*p*: nominal *p*‐value. c) Database for Annotation, Visualization and Integrated Discovery (DAVID) gene ontology pathway analysis of the DEGs. d,e) RT‐qPCR analysis of key gluconeogenic genes in the F1 liver in the refeeding condition. f,g) Alanine tolerance test (ATT) in the presence of vehicle or S3483, an inhibitor of the G6pase system, *n* = 8 mice. Data are mean ± S.E.M. * *p* < 0.05 by *t*‐test or two‐way ANOVA with Holm–Sidak's posthoc.

RNA‐seq analysis of iAsF1‐F and conF1‐F livers found several differentially expressed genes (DEGs) that are enriched in metabolic processes (**Figure** **3**a–c). Particularly, G6pc and Pck1 are rate‐limiting enzymes of gluconeogenesis and direct downstream targets of FoxO1.^[^ ^31^ ^]^ Quantitative reverse transcription PCR (RT‐qPCR) analyses confirmed that G6pc and Pck1 were upregulated in iAsF1‐F liver versus conF1‐F (Figure 3d) but remained unchanged in iAsF1‐M versus conF1‐M (Figure 3e). To test whether the upregulated G6pc is required for the phenotype in iAsF1‐F mice liver, we used S3483, a small molecule inhibitor of the glucose 6‐phosphatase system.^[^ ^32^, ^33^ ^]^ We performed alanine tolerance tests (ATTs) because alanine is one of the major gluconeogenic precursors in vivo. In the absence of S3483, iAsF1‐F mice showed increased glucose production from alanine compared to conF1‐F (Figure 3f), which is consistent with the intolerance to pyruvate and glucose. Such a phenotype was masked by S3483 pretreatment (Figure 3g), demonstrating the essential role of the hepatic FoxO1/G6pc pathway in the paternal iAs effect on glucose homeostasis in the F1 female.

### 2.4 Temporary Growth Retardation in F2 Offspring from Male‐Lineage iAs Exposure

**Figure 4:**
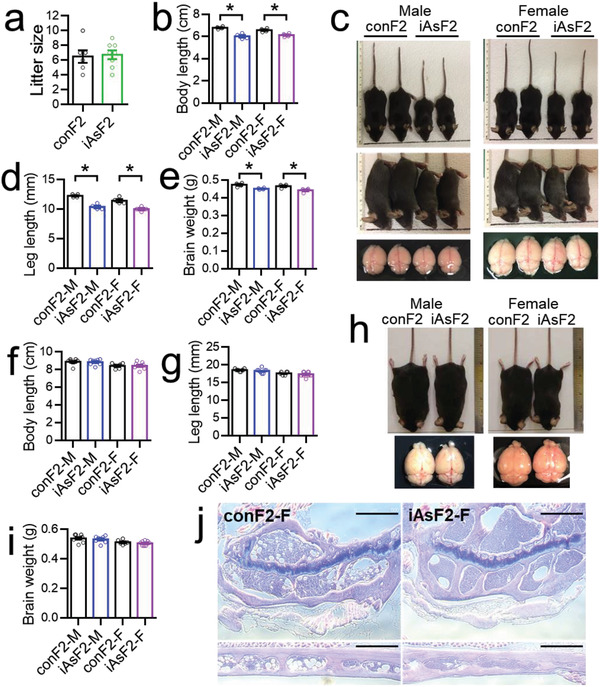
Temporary growth retardation in the F2 offspring from male‐lineage iAs exposure. a) Litter size of the F2 offspring. b–e) Body length, tibia leg length, and brain weight of F2 offspring at the early weaning age (3–4 weeks old). f–i) Body length, tibia leg length, and brain weight of F2 offspring at 12 weeks old. j) H&E staining of the bone at 12 weeks old. Scale bar = 500 µm. Data are mean ± S.E.M. * *p* < 0.05 by *t*‐test.

To address transgenerational effects, we characterized the phenotypic changes in the F2 generation from male‐lineage exposure. Although the litter size was normal (**Figure** **4**a), iAsF2 mice showed small body length (Figure 4b,c), leg length (Figure 4d), and brain weight (Figure 4e) at an early age in both male and female mice, suggesting growth retardation. However, these growth retardations diminished and became insignificant (Figure 4f–i) as the mice grew into adulthood. The bone histology analysis also did not identify any obvious abnormality in adult F1 mice (Figure 4j). Thus, the male‐lineage iAs exposure caused temporary growth retardation in the F2 offspring at a young age.

### 2.5 Energy Imbalance and Nutrient Malabsorption in Adult iAsF2

**Figure 5:**
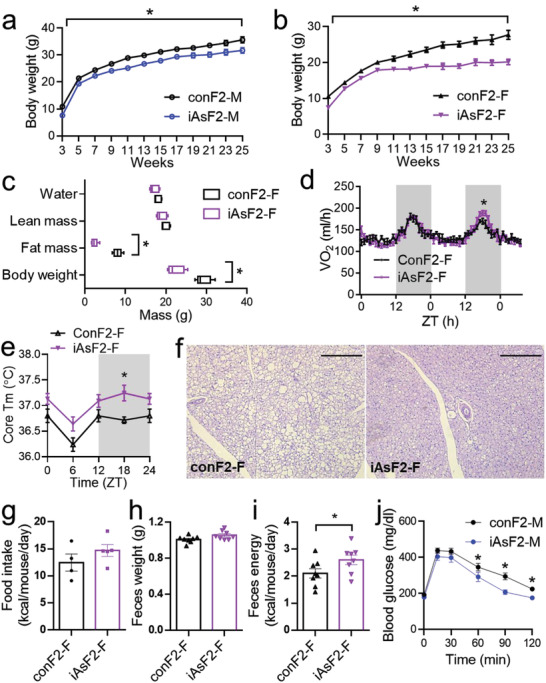
Reduced adiposity and nutrient malabsorption in adult F2 offspring from male‐lineage iAs exposure. a,b) Body weight at the indicated age, *n* = 10 mice. *p < 0.05 by *t*‐test at all timepoints. c) MRI body composition analysis. Box‐plots center lines, limits, and whiskers represent the median, quartile, and minimum/maximum values, respectively, *n* = 5 mice. d) Oxygen consumption by indirect calorimetry. ZT: zeitgeber time. ZT0 = 7 am (light on). ZT12 = 7 pm (light off), *n* = 5 mice. e) Core body temperature measured at the indicated ZT, *n* = 8 mice. f) H&E stain of brown adipose tissue (BAT). Scale bar = 250 µm. g) Food intake in home cages, *n* = 4–5 cages. h) Daily feces weight per mouse, *n* = 8 mice. i) Feces energy content by bomb calorimetry, *n* = 8 mice. j) Glucose tolerance test at 12 weeks old, *n* = 10 mice. Data are mean ± S.E.M. * *p* < 0.05 by *t*‐test or two‐way ANOVA with Holm–Sidak's posthoc.

Although the growth retardation phenotype in the F2 offspring diminished as mice reached adulthood, the body weight remained lower in iAsF2 than conF2 in both male and female mice, and the differences got more profound as mice age (**Figure** **5**a,b). These results suggest that grandpaternal iAs exposure caused a persistent disruption of systemic energy balance in the F2 offspring in both sexes. Body composition analyses with magnetic resonance imaging (MRI) showed that the lower body weight in adult iAsF2 mice was due to lower fat mass but not lean mass (Figure 5c), which is consistent with the normal body length and bone morphology. From the perspective of energy balance, reduced adiposity could be due to reduced energy intake or increased energy output. iAsF2‐F mice showed overall normal energy expenditure with a slight increase in total oxygen consumption at night as measured by indirect calorimetry (Figure 5d). Core body temperature measurement at different times across the day revealed a higher body temperature at night (Figure 5e), which is in line with the slight increase in total energy expenditure at night. Brown adipose tissue (BAT) is a major thermogenic organ in mice. BAT in iAsF2‐F mice showed smaller lipid droplets than conF2‐F mice (Figure 5f), implying a more active thermogenic activity and lipid catabolism. In addition to the energy expenditure, we also measured energy intake. iAsF2‐F mice showed normal daily food intake (Figure 5g). However, food intake is not equal to calorie intake because there may be a difference in the efficiency of food digestion and nutrient assimilation. iAsF2‐F might have nutrient malabsorption, which rendered them less efficient in extracting calories from food. To test this possibility, we monitored daily feces production but found no difference between iAsF2 and conF2 (Figure 5h). We then used bomb calorimetry to analyze the calorie content in the feces. iAsF2 feces showed increased calorie content than conF2 (Figure 5i). It is known that even a tiny error in energy intake and output balance could cause profound changes in body weight in the long run.^[^ ^34^ ^]^ As expected, iAsF2 mice showed improved glucose tolerance than conF2 (Figure 5j), which is likely a result of the reduced adiposity. In summary, these results suggest that an increase in thermogenesis in alliance with nutrient malabsorption may account for the lower adiposity in adult iAsF2 mice.

### 2.6 Fecal Microbiome Changes in Adult iAsF2

**Figure 6:**
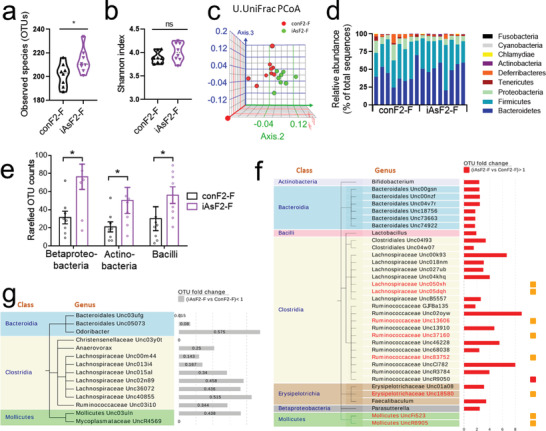
Fecal microbiome changes in adult F2 offspring from male‐lineage iAs exposure, *n* = 8 mice. a) Observed operational taxonomic units (OTUs). b) The *α* diversity. c) Intergroup difference by the Unweighted UniFrac PCoA analysis. d) Relative abundance at the phylum level. e) Comparison at the class level. Data are mean ± S.E.M. f,g) Upregulated and downregulated genera in iAsF2‐F versus conF2‐F. Orange squares: genera only detected in iAsF2‐F. Red square, a genus with a 187‐fold increase in iAsF2‐F versus conF2‐F. * *p* < 0.05 by *t*‐test. ns, not significant.

The gut microbiome plays an important role in nutrient digestion and absorption. The microbiome can have long‐lasting effects on systemic energy homeostasis and is implicated in mediating persistent phenotypic across generations.^[^ ^35^ ^]^ We profiled microbiome composition in iAsF2 and conF2 female mice using 16S ribosomal RNA (rRNA) gene sequencing. The total number of detectable bacterial species was measured by the operational taxonomic units (OTUs) and was higher in iAsF2‐F feces than conF2‐F (**Figure** **6**a). However, the overall diversity of the microbiome was similar between the two groups (Figure 6b). The intergroup difference assessed by the Unweighted UniFrac PCoA analysis revealed that the microbiome of iAsF2‐F had distinct characteristics from that of conF2‐F (Figure 6c). Analyses at the phylum level did not identify noticeable differences (Figure 6d). However, there was a clear difference at the class level. Compared to conF2‐F feces, iAsF2‐F feces were more enriched with the Betaproteobacteria, Actinobacteria, and Bacilli classes (Figure 6e). Some studies suggested that Firmicutes‐to‐Bacteroidetes ratio is lower in lean subjects than obese subjects,^[^ ^36^, ^37^ ^]^ although this is controversial.^[^ ^38^, ^39^, ^40^ ^]^ In our study, the ratio is comparable between conF2‐F and iAsF2‐F (1.055 vs 1.009, *p* = 0.279). At the genus level, several genera were significantly upregulated in iAsF2‐F versus conF2‐F (Figure 6f). Of note, *Ruminococcaceae UncR9050* was upregulated by 187‐fold in iAsF2‐F versus conF2‐F (indicated by the red square), and the other eight genera (indicated by orange squares) were only detectable in iAsF2‐F. There were also a few genera that were significantly downregulated in iAsF2‐F compared to conF2‐F, including those in Bacteroidia, Clostridia, and Mollicutes classes (Figure 6g). It is conceivable that these gut microbiome changes could contribute to nutrient malabsorption and reduced adiposity in iAsF2 mice.

### 2.7 Increased Susceptibility to Diet‐Induced Metabolic Changes in the F3 Offspring

**Figure 7:**
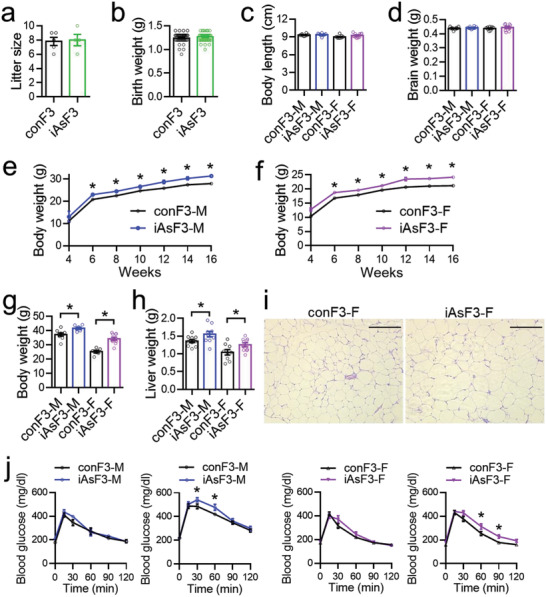
Effects of the male‐lineage iAs exposure on the growth and adiposity of the F3 offspring. a,b) Litter size and birth weight of the F3 offspring. c,d) Body length and brain weight of adult F3 offspring. e,f) Body weight on chow diet for F3 males and females, *n* = 9 mice. g,h) Bodyweight and liver weight after feeding a high‐fat diet (HFD) for a month. i) H&E stain of white adipose tissue (WAT) on HFD Scale bar, 250 µm. j) Glucose tolerance test (GTT) of F3 mice on the indicated diet, *n* = 10 mice. Data are mean ± S.E.M. * *p* < 0.05 by *t*‐test or two‐way ANOVA with Holm–Sidak's posthoc.

We continued to characterize the F3 generation offspring. iAsF3 showed normal litter size and birth weight (**Figure** **7**a,b). Adult iAsF3 mice also displayed normal body length and brain weight (Figure 7c,d). This result demonstrates that the temporary growth retardation observed in F2 was not replicated in F3. Interestingly, iAsF3 showed increased body weight than conF3 as they grow (Figure 7e,f), which was opposite to the lower adiposity phenotype in the F2 offspring. This phenotype was seen in both male and female mice. The body weight phenotype became more obvious when mice were fed with a high‐fat diet (HFD) for a month (Figure 7g), which was associated with increased liver weight (Figure 7h) and bigger adipocytes in the white adipose tissue (Figure 7i). Glucose tolerance tests (GTTs) revealed no significant difference between conF3 and iAsF3 under normal chow diet, but impaired glucose tolerance in iAsF3 on HFD in both sexes (Figure 7j), likely as a result of the increased body weight. These results demonstrate that the male‐lineage iAs exposure increases the susceptibility to diet‐induced obesity and metabolic derangement in the F3 offspring, which is opposite to the F2 offspring. In summary, the findings show that male‐lineage iAs exposure has profound effects on glucose metabolism and energy homeostasis for up to three generations, with distinct impacts on each generation.

## 3 Discussion

The prevalence of metabolic disorders, such as diabetes and obesity, has drastically increased over the past few decades.^[^ ^41^ ^]^ Meanwhile, exposure to synthetic chemical products has also elevated, raising a possibility that environmental chemical pollutants could function as endocrine disruptors or “obesogens” in the pathogenesis of metabolic disorders.^[^ ^42^, ^43^ ^]^ A variety of chemicals, from preservatives to food additives, contain metal or metalloid such as iAs. Here, we systemically analyzed the cross‐generational effects of iAs on metabolic physiology.

A surprising finding from the current study is the sex‐specific effect in the F1 offspring. Female iAsF1 mice, but not males, showed altered glucose tolerance insulin sensitivity. The current evidence suggests hepatic glucose production as the underlying molecular mechanism: 1) female iAsF1 mice were intolerant to insulin as well as gluconeogenic substrates such as pyruvate or alanine as compared to conF1; 2) primary hepatocytes isolated from female iAsF1 mice showed enhanced glucose output than conF1; 3) female iAsF1 liver had a lower glycogen content than conF1 and reduced phosphorylation of FoxO1, a critical regulator of gluconeogenesis; 4) expression of gluconeogenic genes downstream of FoxO1 was altered in female iAsF1 liver; and 5) pharmacologic manipulation of the gluconeogenic enzyme system masked the glucose phenotype in iAsF1 females. However, it is unclear how iAs exposure in F0 males affects F1 metabolism and why such an intergenerational effect is sex specific. DNA methylation, histone modifications, and noncoding RNAs could play roles in the intergenerational effects.^[^ ^8^, ^44^, ^45^, ^46^ ^]^ DNA methylation is maintained by the opposing actions of two classes of enzymes, DNA methyltransferases (DNMTs) and demethylase family Ten–eleven translocations (TETs).^[^ ^47^, ^48^ ^]^ iAs was shown to alter TET activities^[^ ^49^, ^50^ ^]^ and DNA methylation.^[^ ^51^, ^52^, ^53^, ^54^, ^55^, ^56^, ^57^, ^58^ ^]^ DNA methylation has been implicated in the epigenetic inheritance of other environmental exposures, but the causative role of DNA methylation has not been rigorously tested in vivo in mammals.^[^ ^59^, ^60^ ^]^ It is possible that iAs exposure in F0 male alters DNA methylation on the X‐chromosome or imprinted genes in the F0 sperm, which could explain the sex‐specific effect in the F1 offspring. It is also possible that iAs‐induced epigenetic changes do not manifest themselves until they interact with certain sex hormones. Establishing the central role of hepatic glucose production in the F1 female glucose phenotype in the current study provides a solid starting point to further characterize the intergenerational and sex‐specific molecular mechanisms in the future.

A second surprising finding is the presence of a phenotype in F2 offspring despite the lack of an obvious phenotypic change in F1 males. It is likely that male iAsF1 mice had some cryptic phenotypic changes compared to conF1 males, which were not captured in the current study. We focused on glucose metabolism and energy balance in the current study, and did not examine lipid metabolism, the immune system, or the reproduction system. The cryptic phenotypic changes may alter the epigenome of the F1 male germ cells in a different way compared to the direct iAs exposure on the F0 sperm. These epigenetic changes then translate into gene expression alterations in the F2 offspring at multiple tissues or organs, leading to temporary growth retardation at a young age and altered metabolites in the gut. The change in the gut microbiome is likely an outcome of the altered metabolites since different bacterial species have different proliferation rates when feeding on different metabolites. The altered microbiome may contribute to nutrient malabsorption and reduced adiposity in adults. The effect of direct arsenic exposure on gut microbiome was studied before.^[^ ^61^ ^]^ In six week old infants, 8 genera from *Firmicutes* phylum and 15 genera from other phyla were positively or negatively correlated with urinary arsenic levels.^[^ ^62^ ^]^ Arsenic treatment also changed the taxonomic profile of the microbiome in murine models.^[^ ^63^, ^64^ ^]^ Here we demonstrate an indirect, transgenerational effect of paternal arsenic exposure on gut microbiome at the F2 generation. We do not think that microbiome changes can be inherited across generations in this male‐lineage paradigm. Rather, we speculate that the microbiome changes connect the upstream epigenetic alterations with the downstream energy imbalance. In addition to the microbiome‐mediated nutrient malabsorption, altered activity in the brown adipose tissue thermogenesis and increased energy expenditure may also contribute to the reduced adiposity in the adult F2 offspring.

A third surprising finding is the opposite phenotypes in F2 versus F3 offspring. The F2 offspring showed reduced adiposity, while the F3 offspring showed higher adiposity, especially in the presence of a high‐calorie diet. It is possible that the growth retardation and nutrient malabsorption in the iAsF2 male mice elicit “thrifty” adaptations on the epigenome of their germ cells, which gets transmitted into the F3 generation. This notion is in keeping with the thrifty phenotype hypothesis,^[^ ^65^ ^]^ but with a cross‐generational implication.

There is some limited epidemiologic evidence linking early‐life iAs exposure with later‐life metabolic disorders.^[^ ^57^, ^66^, ^67^ ^]^ The interpretation of human epidemiologic data can be confounded by many factors. Many regions with iAs‐contaminated drinking water have been inhabited by humans over several generations. The outcome of iAs exposure is a mixture of the short‐term acute exposure and long‐term effects due to early‐life exposure that may impact prenatal and postnatal developmental processes.^[^ ^23^, ^24^, ^25^, ^26^, ^27^, ^28^, ^29^ ^]^ Our study demonstrates robust intergenerational and transgenerational effects of male‐lineage iAs exposure in the mammal for the first time. The distinct effects on different generations or sexes found in our study unveiled layers of complexity that are previously unknown in iAs toxicity.

The paternal effects of dietary factors or psychological stress on F1 offspring metabolism have been reported.^[^ ^9^, ^10^, ^11^, ^12^ ^]^ However, the transgenerational effects across multiple generations remain unclear. Exposures of gestating females to endocrine‐disrupting compounds have been shown to affect adiposity in offspring of multiple generations, although it is unclear whether it is transmitted through the male germline, female germline, a combination of both germlines, or other mechanisms.^[^ ^60^, ^68^ ^]^ It has also been challenging to pinpoint the organ or cell type in the offspring as the major contributor to the metabolic phenotype. The current findings of the transgenerational effects of iAs through the exclusive male‐lineage transmission shed light on some of these issues and unveiled a few fascinating phenomena in the cross‐generational effects of environmental exposure on metabolism. 1) The cross‐generational effect does not always diminish as it is transmitted through multiple generations. The low dose and short duration of the iAs exposure in the current study did not cause glucose intolerance in adult F0 males but produced robust changes in glucose tolerance in F1 offspring. Likewise, iAsF0 and iAsF1 mice had normal body weight, but iAsF2 and iAsF3 offspring showed abnormal body weight. 2) The transgenerational effects do not always go in the same direction across generations. Ancestral exposure to iAs decreased adiposity in F2 offspring but increased adiposity in F3 offspring. These results suggest that an environmental chemical or endocrine‐disrupting compound might suppress adiposity in a generation‐specific manner, rather than always functioning as an obesogen. 3) Ancestral environmental exposure can disrupt metabolism without causing obesity. iAsF1 females showed glucose intolerance and insulin resistance despite normal body weight. These results suggest that ancestral exposure can create a cryptic epigenetic predisposition to prediabetes or diabetes without affecting body weight. 4) Ancestral environmental exposure can disrupt metabolism in a highly sex‐specific manner. iAsF1 males did not show glucose intolerance, while iAsF1 females did. 5) The organ or cell type that mediates the metabolic effects of ancestral exposure can be defined in adult offspring. We pinpointed the liver and hepatocyte gluconeogenesis as the major contributor to the glucose intolerance phenotype in iAsF1 females through comprehensive metabolic testing, cell‐autonomous assays, and pharmacologic rescue experimentation. Future mechanistic studies using the iAs male‐lineage transmission as a model are likely to provide further insights into the cross‐generational effects of environmental factors on metabolism.

## 4 Experimental Section

##### Mouse Housing and iAs Treatment

C57BL/6 male and female mice were housed and bred under pathogen‐free conditions. Mice were group‐housed with 3–4 mice per cage at 22–25 °C and 40–70% humidity under the standard 12 h light/12 h dark cycles. Mice were fed with a normal chow diet (LabDiet #5015) or a diet containing 60 kcal% fat (ResearchDiets # D12492). iAs content in the diet was not indicated by the manufacture on the product sheet. The iAsF0‐M mice were treated with 250 ppb iAs in the drinking water for 3 weeks, starting at around the age of 8 weeks old. Sodium arsenite solution was prepared by dissolving the sodium arsenite powder (Sigma–Aldrich, purity ≥ 99%) in deionized water. As described previously,^[^ ^69^ ^]^ mice exposure experiments were performed in the units specialized for environmental toxicity exposure research. iAs solution was diluted with clean drinking water to the final concentration of 250 ppb. All the drinking water was prepared freshly and refilled weekly. Mouse breeding was conducted in the standard mouse housing suites. All experimental procedures were approved by the Institutional Animal Care and Use Committee at Baylor College of Medicine.

##### Measurement of Energy Expenditure, Food Intake, and Body Composition

Energy expenditure and food intake were monitored by the Comprehensive Laboratory Animal Monitoring System (CLAMS) equipped with the Oxymax indirect calorimetric assessment (Columbus Instruments, OH). Mice were prehoused in the unit for at least 24 h for habituation before continuous data collection for 48 h under normal light–dark schedules. Body composition analysis was performed using the EchoMRI nuclear magnetic resonance (NMR) relaxometry (EchoMRI L.L.C., TX). These analyses were done at the Mouse Metabolic and Phenotyping Core (MMPC) in Baylor College of Medicine. Core body temperature was determined by a rectal thermometer probe (Physitemp Instruments, NJ).

##### Feces Collection and Bomb Calorimetry

For feces collection, mice were single‐housed in metabolic cages equipped with urine and feces collection units. All the equipment was autoclaved and dried. Mice were allowed to habituate in the presence of clean water and food pellets for at least 24 h. Fecal samples were obtained in the original form without contamination by urine, water, fur, or food crumbs. Fresh feces were transferred to clean Eppendorf tubes every 8 h followed by storage at −80^ ^°C. Digestible energy content in feces was measured using a bomb calorimeter at the Small Animal Phenotyping Core facility at the University of Alabama at Birmingham.

##### Kinetic Metabolic Testing and Serum Insulin Measurement

Kinetic metabolic testings performed in the current study include GTT, insulin tolerance test (ITT), pyruvate tolerance test (PTT), and ATT. Mice fasted for 6–9 h. A bonus of 2 g kg^−1^ glucose (d‐(+)‐glucose, Sigma–Aldrich), 0.75 mIU kg^−1^ insulin (Humulin, Eli Lily), 2 g kg^−1^ pyruvate (Sigma–Aldrich), or 4 g kg^−1^ alanine (Sigma–Aldrich) was administered via intraperitoneal injection, and then blood glucose concentration was monitored through tail vein bleeding and glucometer (OneTouch Ultra 2, Lifescan) across a time course. Mice were allowed to recover for 2 weeks in between different tests. For the test involving S3483, mice were intraperitoneal injected with 50 mg kg^−1^ S3483 or vehicle and allowed to rest for 10 min. The blood glucose level was measured, followed by alanine injection, blood sampling, and blood glucose measurement, as described above. Insulin levels in serum samples were determined using the ultrasensitive mouse insulin enzyme‐linked immunosorbent assay (ELISA) kit (#90 080, Crystal Chem) following the manufacture's instruction.

##### Primary Hepatocyte Isolation and Glucose Output Assay

Primary hepatocytes were isolated from adult mice via a modified two‐step perfusion method.^[^ ^70^ ^]^ Primary hepatocytes were further purified with centrifugation in the presence of Percoll (Amersham Biosciences AB, Piscataway, NJ) and cultured in collagen‐coated plates in Dulbecco′s modified Eagle′s medium with 10% fetal bovine serum (FBS) for 4 h until attachment. Cells were then starved in glucose output media (118 × 10^−3^ m NaCl, 4.7 × 10^−3^ m KCl, 1.2 × 10^−3^ m MgSO_4_, 1.2 × 10^−3^ m KH_2_PO_4_, 1.2 × 10^−3^ m CaCl_2_, 20 × 10^−3^ m NaCO_3_, 20 × 10^−3^ m 4‐(2‐Hydroxyethyl)piperazine‐1‐ethanesulfonic acid (HEPES), and pH 7.4) for 1 h before treated with different substrates including lactate (10 × 10^−3^ m lactate and 1 × 10^−3^ m pyruvate), glycerol (5 × 10^−3^ m), or glutamine (5 × 10^−3^ m). After 2 h, an aliquot of glucose output media was taken for measurement of glucose using Glucose (Hexokinase) Liquid Reagents kit (#G7517120, Fisher Scientific). The data were normalized to the total protein content of the cell lysates.

##### Histology

Fresh liver tissue, white adipose tissue, and brown adipose tissues were fixed in 10% formalin overnight and embedded in paraffin. Sections were cut at a thickness of 5 µm and stained using the hematoxylin and eosin (H&E) or periodic acid–Schiff (PAS) method. For bone tissues, decalcification and hydration were done before paraffin embedding. Sections were imaged and analyzed by microscopy (Leica Microsystems, Wetzlar, Germany).

##### Body Length, Leg Length, and Brain Weight Measurement

These measurements were performed on euthanized mice. For body length, mice were placed in a prone position, measuring from the tip of the nose to the base of the tail. The tibia leg length was measured from knee to ankle. The whole brain was dissected out and weighed on the scale.

##### Protein Extraction and Western Blot

Liver samples were harvested after refeeding for 5 h following overnight fasting. Liver samples were dissected and frozen immediately in liquid nitrogen. The liver samples were homogenized in radioimmunoprecipitation assay (RIPA) buffer supplemented with proteinase inhibitor cocktails (Santa Cruz Biotech), phosphatase inhibitors (Sigma–Aldrich), and phenylmethylsulfonyl fluoride (PMSF) (EMD Millipore #52 332). Protein concentration was measured using Bradford protein assay according to the manufacturer's instructions (Bio‐Rad). For western blot analysis, total protein lysates were resolved on sodium dodecyl sulphate‐polyacrylamide gel electrophoresis (SDS‐PAGE), transferred to polyvinylidene fluoride (PVDF) membrane, and blotted with primary antibodies including Phospho‐AKT (Ser473) (CST 736E11, #3787), total AKT (CST #9272), Phospho‐FoxO1 (Ser256) (CST #9461), total FoxO1 (CST C29H4, #2880), and Hsp90 (CST #4874) and horseradish peroxidase (HRP)‐conjugated secondary antibodies (Santa Cruz Biotech sc‐516102 or sc‐2357).

##### RT‐qPCR and RNA‐seq

Total RNA was extracted from frozen liver samples using Trizol reagent (LifeTech) and was further purified using the RNeasy Mini Kit (Qiagen). RT‐qPCR was processed using the High‐Capacity cDNA Reverse Transcription Kit (Applied Biosystems), PowerUp SYBR Green Master Mix (Thermo Fisher), and QuantStudio 6 instrument (Thermo Fisher). Standard curves were generated by series dilution of pooled RNA samples. The relative value for each gene was normalized to the 18S RNA of the same sample as the housekeeping control. For RNA‐seq, RNA‐seq library preparation and sequencing were performed at the UCLA Technology Center for Genomics & Bioinformatics (TCGB) Core facility. Libraries were sequenced on the Illumina HiSeq 2000/2500 platform. Raw reads from biological triplicates were analyzed as previously described.^[^ ^69^ ^]^ Functional analysis of differentiated expression genes was performed using GSEA software (V4.0.3).^[^ ^71^ ^]^

##### Microbiome 16S rRNA Gene Sequencing and Analysis

Fecal samples were obtained directly from the anus without contamination with urine or fur. The fecal samples were collected in pathogen‐free microcentrifuge tubes and stored at −80 °C until processing. 16S rRNA gene sequencing was performed in the core facility of Alkek Center for Metagenomics and Microbiome Research at Baylor College of Medicine. Observed OTUs indicated overall microbiome diversity in test samples. The Shannon index was calculated for assessing the *α* diversity, a measurement of the variety of organisms detected in each group. These two indicators described the general enrichment of different groups. Unweighted principal coordinates analysis (PCoA) analysis was used to assess intergroup differences. Classification of reads by taxonomic levels provides more detailed information. Different bacteria are clustered from phylum through genera.

##### Statistical Analysis

No statistical methods were used to predetermine sample sizes. Instead, sample sizes were determined based on previous publications for each assay. Normality was tested by the Shapiro–Wilk test. Data were not preprocessed except that RT‐qPCR data were normalized to the housekeeping genes. All measurements were taken from distinct biological samples (mice or litter). Most comparisons between two groups were analyzed using a two‐tailed unpaired *t*‐test. Kinetic metabolic tests with multiple time points were analyzed by two‐way repeated‐measures analysis of variance (ANOVA) with Holm–Sidak's posthoc test. For 16S rRNA gene sequencing, independent samples’ Mann–Whitney U test was used. All tests were two sided. Most data were expressed as mean ± standard error of mean (S.E.M.) unless otherwise indicated in the figure legend. The minimal level of significance was set at *p* < 0.05. Sample size and statistical methods for each test were provided in figure or/and figure legends. Most key animal experiments were performed twice using independent biological samples, except the RNA‐seq studies that were performed once. Experimenters were bound to the genotype. Data were excluded using the pre‐established criteria. Animals were excluded before metabolic tests if they showed distress, infection, bleeding, or anorexia. SPSS 22.0, Microsoft Excel 2010, Prism Graphpad 7 were used for statistical analysis and visualization.

##### Data Accession

RNA‐seq data were uploaded to Gene Expression Omnibus (GSE154130).

## Conflict of Interest

The authors declare no conflict of interest.

## Author Contributions

Y.G. and Y.X. contributed equally to this work. Z.S. and G.D. conceived the study. Y.G., Y.X., X.L., W.Z., and G.D. collected data. P.M. and A.B. provided reagents. Y.G., Y.X., X.L., W.Z., G.D., Z.Z., L.H., and Z.S. analyzed data. Y.G., Y.X., G.D., and Z.S. interpreted the data. S.M. supported Y.X. Z.S. acquired funding. Y.G. and Z.S. wrote the manuscript with input from the other authors.
